# Dendritic Cell-Mediated Regulation of Liver Ischemia-Reperfusion Injury and Liver Transplant Rejection

**DOI:** 10.3389/fimmu.2021.705465

**Published:** 2021-06-28

**Authors:** Ryosuke Nakano, Lillian M. Tran, David A. Geller, Camila Macedo, Diana M. Metes, Angus W. Thomson

**Affiliations:** ^1^ Department of Surgery, Starzl Transplantation Institute, University of Pittsburgh School of Medicine, Pittsburgh, PA, United States; ^2^ Liver Cancer Center, University of Pittsburgh Medical Center, Pittsburgh, PA, United States; ^3^ Department of Immunology, University of Pittsburgh School of Medicine, Pittsburgh, PA, United States

**Keywords:** liver, dendritic cells, ischemia-reperfusion injury, immune regulation, transplant tolerance

## Abstract

Liver allograft recipients are more likely to develop transplantation tolerance than those that receive other types of organ graft. Experimental studies suggest that immune cells and other non-parenchymal cells in the unique liver microenvironment play critical roles in promoting liver tolerogenicity. Of these, liver interstitial dendritic cells (DCs) are heterogeneous, innate immune cells that appear to play pivotal roles in the instigation, integration and regulation of inflammatory responses after liver transplantation. Interstitial liver DCs (recruited *in situ* or derived from circulating precursors) have been implicated in regulation of both ischemia/reperfusion injury (IRI) and anti-donor immunity. Thus, livers transplanted from mice constitutively lacking DCs into syngeneic, wild-type recipients, display increased tissue injury, indicating a protective role of liver-resident donor DCs against transplant IRI. Also, donor DC depletion before transplant prevents mouse spontaneous liver allograft tolerance across major histocompatibility complex (MHC) barriers. On the other hand, mouse liver graft-infiltrating host DCs that acquire donor MHC antigen *via* “cross-dressing”, regulate anti-donor T cell reactivity in association with exhaustion of graft-infiltrating T cells and promote allograft tolerance. In an early phase clinical trial, infusion of donor-derived regulatory DCs (DCreg) before living donor liver transplantation can induce alterations in host T cell populations that may be conducive to attenuation of anti-donor immune reactivity. We discuss the role of DCs in regulation of warm and liver transplant IRI and the induction of liver allograft tolerance. We also address design of cell therapies using DCreg to reduce the immunosuppressive drug burden and promote clinical liver allograft tolerance.

## Introduction

### Dendritic Cell Biology and Diversity

Our understanding of DC development and function is based largely on extensive studies in mouse models and human *in vitro* systems. DCs are heterogeneous innate immune cells that link innate and adaptive immunity (1). They are subdivided into conventional DCs (cDCs) that acquire, process and present antigen (Ag), and non-conventional plasmacytoid DCs (pDCs) that produce type-1 interferon (IFN) following viral stimulation. While indispensable for antiviral immunity, pDCs also promote or regulate other inflammatory/immune responses (2, 3). cDCs and pDCs arise from a common bone marrow precursor in a fms-like tyrosine kinase 3 ligand (Flt3L)-dependent manner (4–7). Mouse cDCs are further divided into two subsets,- cDC1 (CD11c+,CD103+,CD11b-) and cDC2 (CD11c+,CD103-,CD11b+) that differentiate under the influence of IFN regulatory factor (IRF) 8 and IRF4, respectively. Mouse pDCs are CD11c+, CD103-, CD11b-,B220+,Gr-1+, sialic acid-binding immunoglobulin-like lectin (Siglec) H+ (5, 6, 8–11). Human cDCs express high levels of CD11c and are subdivided into CD1c+ (blood DC Ag (BDCA)1+), CD1b+, CD11b+, CD14+ DC that promote T helper (Th)17 cells and correspond broadly to mouse cDC2s, versus CD141+ (BDCA3+) DC that promote Th1 cell responses and Ag cross-priming to CD8+ T cells, corresponding to mouse DC1s. Human pDCs express CD123 (IL-3R), CD14 and CD303 (BDCA2) and potently produce type-1 IFN. Each DC subset in mice and humans develops under the control of a specific repertoire of transcription factors involving differential levels of IRF8 and IRF4 expression (12–16).

### Liver Dendritic Cells

Multiple DC subsets have been identified in the liver, although their relative abundance differs from that in peripheral blood and secondary lymphoid tissue (17–21). cDC1, cDC2 and pDCs and their functional relevance in the steady-state and liver disease have been reviewed (21). Improved understanding of liver DC heterogeneity and function in mice and humans is required to further elucidate their roles and for design of DC-directed therapeutic intervention in liver injury, transplantation and other liver disorders. Recently, single cell RNA sequencing (seq) analysis has been used to quantify liver DC subsets (cDC1, cDC2 and pDC) and to define signatures of DC-T cell interactions in draining lymph nodes under healthy conditions and in liver disease (22). Mouse liver DC heterogeneity has also been described using cellular indexing of transcriptomes and epitopes by sequencing (CITE-seq) (23). The phenotype and function of liver interstitial DCs is influenced by the hepatic microenvironment that promotes their inherent tolerogenicity in the healthy steady-state (24–26). Thus, *via* their production of macrophage colony-stimulating factor and other soluble and cell-cell contact factors, liver stromal cells induce regulatory cDCs that secrete high levels of IL-10 and nitric oxide (NO), but little IL-12 and inhibit T cell proliferative responses/induce activated T cell apoptosis (24, 27, 28). Exposure to gut-derived pathogen-associated microbial products e.g. bacterial lipopolysaccharide (LPS) inhibits liver cDC or pDC maturation by stimulating IL-6- signal transducer and activator of transcription 3 (STAT3) activity that upregulates expression of interleukin-1 receptor-associated kinase M (IRAK-M), an inhibitor of Toll-like receptor (TLR) signaling (29). This phenomenon, referred to as endotoxin tolerance (30), extends to several TLRs (cross-tolerance), as well as to TLRs and ischemic injury. By contrast, exposure to LPS stimulates secretion of IL-10 and IL-27 by liver cDCs that can then expand regulatory T cells (Tregs) (31, 32).

Liver cDCs also express comparatively low levels of major histocompatibility complex (MHC) class II and co-stimulatory molecules (33), but comparatively high levels of the T cell co-inhibitory molecule programed death ligand -1 (PD-L1). Compared with lymphoid tissue DCs, they also express high levels of the ectoenzyme CD39 (34) that degrades adenosine triphosphate to adenosine, and the immunoreceptor transmembrane adaptor protein DNAX activating protein of 12 kDa (DAP12) that regulates their maturation (35). Like liver cDCs, liver pDCs express comparatively high levels of DAP12 and high PD-L1:CD80/86 ratios (36, 37) and secrete IL-10. Thus, liver DCs are refractory to stimulation with microbial products and express gene products that undermine effector T cell responses, but promote Tregs (38).

We discuss below reported roles of liver DCs in regulation of liver IRI and immune responses to liver allografts. We also consider how regulatory DC (DCreg) therapy is being introduced in clinical trials to ascertain its potential to promote reduced dependency on/withdrawal of immunosuppression (IS) in liver transplantation.

## Liver Ischemia Reperfusion Injury (IRI)

Graft IRI remains an understudied area in transplantation, despite its clinical significance. Hepatocellular damage associated with liver removal, storage and engraftment is critical to primary graft non-function or late dysfunction and may promote acute and chronic rejection and graft loss (39–41). IRI is a complex process that occurs when hypoxic tissue damage is increased by the inflammatory pathways that are activated during the return of blood flow and oxygen delivery, that combines elements of “warm” and “cold” injury (39, 42). Warm IRI is dominated by liver macrophage-derived cytotoxic molecule-mediated hepatocellular damage. Cold IRI, that occurs during ex-vivo organ preservation, is dominated by damage to liver sinusoidal endothelial cells (SECs) and disruption of the microcirculation (39, 43, 44).

### Liver DCs and Regulation of Liver IRI

Regulatory properties of liver DCs have been described in both liver warm and cold (transplant) IRI in the mouse (**Table 1**).

**Table 1:** Regulation of liver ischemia-reperfusion injury by intra-hepatic dendritic cells.

Model(species)	Observation	Protective or deleterious effect of DCs	Reference
Warm IR (mouse)	IR results in enhanced expression of anti-inflammatory cytokines (IL-10; TGFb) but reduced expression of IL-12 by liver DCs	Protective	Loi et al. (45)
Warm IR (mouse)	Targeted deletion of cDCs increases liver injury; cDCs reduce liver IRI *via* IL-10 secretion	Protective	Bamboat et al. (46)
Warm IR (mouse)	VitD analogue administration promotes tolerogenic DCs and attenuates liver injury; interruption of DC-T cell interaction (with anti-CD44) increases proinflammatory DC maturation and enhances tissue damage	Protective	Funken et al. (47)
Warm IR (mouse)	Adoptive transfer of WT but not DAP12-/- DCs reduces liver IRI in DAP12-/- mice *	Protective	Nakao et al. (48)
Warm IR (mouse)	EP3-expressing DCs orchestrate the pro-reparative environment during liver repair after hepatic IR	Protective	Nakamoto et al. (49)
Warm IR (mouse)	Increasing liver DCs in WT but not in TLR4 KO mice promotes liver injury	Deleterious	Tsung et al. (50)
Warm IR (mouse)	Liver injury less in DC-deficient (Flt3L -/-) mice	Deleterious	Zhang et al. (51)
Warm IR (mouse)	Blockade of TIM-4 on hepatic DCs ameliorates liver injury	Deleterious	Li et al. (52)
Warm IR (mouse)	pDC-depleted mice display reduced liver IR injury	Deleterious	Castellaneta et al. (53)
Liver transplant IR (mouse)	Livers from DC-deficient (Flt3L -/-) donors exhibit enhanced injury	Protective	Zhang et al. (51)
Liver transplant IR (mouse)	Portal venous delivery of WT but not CD39-/- liver cDCs to donor livers protects against graft injury	Protective	Yoshida et al. (34)

cDC, conventional DC; DAP12, DNAX activating protein of 12kDa; EP3, E prostanoid receptor 3; Flt3L, fms-like tyrosine kinase 3 ligand; pDC, plasmacytoid DC; TGFb, transforming growth factor b; TIM-4, T cell Ig domain and mucin domain 4; vitD, vitamin D; WT, wild-type.

*DAP12-/- mice exhibit enhanced liver warm IRI compared with WT mice.

#### Liver Warm IRI

Loi et al. (45) reported that liver DCs isolated after hepatic warm IR exhibited a more mature surface phenotype than those from uninjured liver, but preferentially produced the anti-inflammatory cytokines IL-10 and transforming growth factor b that might inhibit T cell and natural killer (NK) cell stimulation after IRI. It was also shown (46) that targeted deletion of cDCs by injecting CD11c-diptheria toxin (DT) receptor mice with DT 12–18 hours prior to I/R increased liver injury. Moreover, cDCs reduced liver IRI by secreting IL-10 that inhibited IL-6, tumor necrosis factor (TNF) and reactive oxygen species production by inflammatory monocytes recruited to the liver. More recent work (49) indicates that signaling *via* the prostaglandin E receptor EP3 in DCs promotes liver repair after warm IR by inducing IL-13-mediated switching of macrophages from pro-inflammatory to IL-10-producing, reparative cells. Vitamin D analogue administration promotes regulatory DCs and attenuates liver warm IRI, whereas interruption of DC-T cell interaction enhances proinflammatory DC maturation and tissue damage (47). Adoptive transfer of wild-type (WT) but not DAP12-/- cDCs reduces warm liver IRI in DAP12-/- mice that exhibit enhanced tissue injury compared with WT animals (48). Taken together, these findings suggest a protective role for DCs in warm liver IRI.

Other data however, conflict with this view. Fms-like tyrosine kinase 3 ligand (Flt3L) is a potent, endogenous DC poietin. Flt3L KO mice exhibit profound reductions in mDC and pDC in liver and lymphoid tissues (51, 54, 55). In these Flt3L KO mice (51), warm liver IR results in reduced hepatic injury, with less polymorphonuclear cell infiltration compared with WT animals. Absence of hepatic interstitial DC in this study also induces less upregulation of inflammatory cytokine and chemokine (TNFa, CCL2 and CXCL2) gene expression in the liver. Moreover, adoptive transfer of splenic or hepatic WT DC into Flt3L KO or WT mice increases hepatic warm IR injury. TIM-4 (T cell immunoglobulin domain and mucin domain containing 4) expression by liver cDC has been reported to play an important role in mouse segmental warm IRI (52); its blockade by anti-TIM-4 antibody reduces liver injury and inflammatory cytokine production and facilitates induction of Foxp3+ Tregs, suggesting a potential therapeutic approach. Thus, in contrast to the protective roles of liver DC described above, these findings suggest injurious effects of DC in liver warm IRI (51). In another report (50), increasing cDCs in the liver by GM-CSF hydrodynamic transfection increased liver injury after warm IR in WT but not TLR4 KO mice. With respect to liver pDCs, mice depleted of these cells using anti-pDC Ag (PDCA)-1 antibody failed to upregulate hepatic IFNa and exhibited reduced levels of hepatic IL-6, TNFa and liver injury after warm IR compared with WT controls (53).

Thus, while reports using different experimental approaches suggest both protective and deleterious effects of liver DCs in liver warm IRI, the balance of reports indicate protective properties of these cells in mouse models (53, 56–59). Further studies, taking into account liver DC heterogeneity and focused on the role of specific hepatic DC subsets, as well as the release of small extracellular vesicles with proinflammatory versus reparative properties by these cells during warm IRI (60, 61), may help elucidate these conflicting observations. Depending on microenvironmental conditions, complement system activators and inhibitors may also influence the differentiation/function of DC subsets towards immunogenicity or tolerance (62) and may be worthy of further investigation in the context of liver DCs their regulation of hepatic inflammatory responses.

#### Liver Transplant IRI

Several molecules have been implicated in regulation of liver transplant (cold) IRI by DCs. Absence of CD39 in liver grafts enhances cold IRI, associated with higher levels of pro‐inflammatory cytokines (IL-6, TNFa, monocyte chemoattractant protein-1, IL-12p40), compared to WT donors. In addition, these CD39-/- allografts express higher levels of DC maturation markers (CD80, CD86, MHC II) and lower levels of coinhibitory PD-L1. Moreover, adoptive transfer of WT liver mDCs exerts a protective effect against transplant-induced liver IRI, that is not achieved by CD39-/- liver mDC infusion (34, 63).

When Flt3L KO donor livers (lacking interstitial DCs) are transplanted into syngeneic WT mice with 24 hours of cold ischemia, the grafts show dramatically increased IR injury, with enhanced alanine transaminase levels, hepatic necrosis and neutrophil infiltration, indicating a protective role of liver-resident DC in WT livers (51). Thus, from the limited studies undertaken to date, liver DCs appear to have a protective role against liver transplant IRI in mice.

## Liver Transplant Tolerance

The liver is considered a tolerogenic environment, as evidenced by oral tolerance, portal venous tolerance, the ability of adeno-associated viral gene therapy to induce systemic tolerance to a transgene (64), metastasis of tumors to the liver and, in animals, acceptance of liver allografts across MHC barriers, without IS therapy (65–69). Within the liver microenvironment, multiple parenchymal and non-parenchymal cell populations (including DCs, Kupffer cells, SECs and stellate cells) express gene products e.g. indoleamine dioxygenase, arginase and PD-L1, that suppress inflammatory and immune-mediated responses (70). DCs express human leukocyte Ig-like receptor B (LILRB) family members, ligation of which renders DCs tolerogenic, leading in turn, to suppression of T cell responses (71) and immune tolerance in humanized mice. Since LILRB family members are considered receptors for HLA-G, that can be produced by liver cells (hepatocytes, liver stem/progenitor cells and biliary epithelial cells) (27, 72–74), this may potentially be an additional mechanism of immune regulation within the liver environment. The liver is also considered a site in which T cells activated therein exhibit defective cytotoxic function (75), and a site of increased T cell apoptosis (76). Potential mechanisms that may mediate liver transplant tolerance have been reviewed recently (26, 77).

### Liver DCs and Regulation of the Balance Between Liver Transplant Tolerance and Rejection

Hematopoietic progenitors within the liver are programed to differentiate into DCregs with comparatively low MHC II and T cell costimulatory molecule expression and high IL-10 but low IL-12 secretion. Both liver cDCs and pDCs only weakly stimulate allogeneic T cell proliferation and can promote activated T cell hyporesponsiveness/apoptosis and Tregs (32, 78–80). Together with other liver NPCs, liver DCreg appear to play key roles in the induction of liver transplant tolerance (24); reviewed in (25, 26, 70, 81). The properties of mouse and human hepatic DCs that may promote regulation of alloreactive T cell responses/tolerance induction are summarized in **Table 2**.

**Table 2:** Properties of hepatic DCs that promote their immune regulatory function and may contribute to tolerance induction.

DC subset (species)	Property	Effect	Reference(s)
**cDCs**			
Mouse	Low MHC class II and costimulatory molecule expression	Infusion into prospective pancreatic islet allograft recipients prolongs graft survival	Rastellini et al. (38)
Mouse	Low MHC class II and co-stimulatory molecule expression	Infusion induces IL-10-producing cells in allogeneic host lymphoid tissue	Khanna et al. (33)
Mouse	Low MHC class II and costimulatory molecule expression	Systemic administration induces donor-specific T cell hyporesponsiveness in a sponge allograft model	Chiang et al. (82)
Mouse	Low TLR4 expression	Induction of alloAg-specific T cell hyporesponsiveness following LPS stimulation	Dr Creus et al. (83)
Mouse (also pDCs)	Gut-derived bacterial products inhibit liver DC maturation by stimulating IL-6-STAT3 activity that upregulates IRAK-M expression	Higher maturation marker expression by IL-6 -/- liver cDCs and pDCs	Lunz et al. (29)
Human	Production of IL-10 but not IL-12p70, even after TLR4 stimulation	Poor ability to stimulate allogeneic T cell proliferation; stimulation of IL-10 but suppression of IFNg production by T cells	Goddard et al. (84); Kwekkeboom et al. (85)
Human	Liver perfusate DCs exhibit low costimulatory molecule expression and produce high IL-10 levels in response to TLR4 ligation	Impaired T cell stimulatory capacity compared with skin or secondary lymphoid tissue DCs	Bosma et al. (86)
Mouse	Periportal and sinusoidal liver DCs loaded with Ag in the portal vein	Induce Th2 responses in the liver, enhance apoptosis of Ag-specific T cells and prevent hepatic injury caused by Th1 cells.	Watanabe et al. (87)
Mouse	Reduced costimulatory molecule and IL-12 expression induced by contact with sinusoidal endothelial cells	Impaired ability to prime naïve CD8 T cells	Schildberg et al. (88)
Mouse	IL-10 production; low Delta 4/Jagged 1 Notch ligand ratio	Skew towards allogeneic Th2 cell differentiation; CD4 T cell apoptosis; poor T cell allostimulatory activity associated with Treg function	Tokita et al. (79)
Mouse	Liver stromal cell-induced DCs secrete high IL-10/low IL-12; produce PGE2	Inhibit T cell proliferation/induce apoptosis of activated T cells; alleviate autoimmune hepatitis	Xia et al. (24)
Human	Liver stromal cells impair DC differentiation and maturation (role for PGE2)	Impaired ability to induce T cell proliferation	Bruno et al. (27)
Mouse	Liver stroma induces regulatory DCs producing NO and IL-10	Inhibition of CD8 T cell proliferation	Wang et al. (28)
Human	Secrete substantial IL-10 upon TLR4 ligation	Generate more suppressive Tregs than blood DCs *via* an IL-10-dependent mechanism	Bamboat et al. (32)
Mouse	LPS-stimulated liver DCs secrete IL-10 and IL-27	Induce T cell hyporesponsiveness, associated with selective Treg expansion	Chen et al. (31)
Mouse & Human	Liver DCs with low lipid levels	Induce regulatory T cells, anergy to cancer, and oral tolerance	Ibrahim et al. (89)
Mouse	Comparatively high cell surface CD39 expression	Hyporesponsiveness to ATP; reduces responses to TLR4 ligation and proinflammatory and immunostimulatory activity	Yoshida et al. (34)
Mouse	Absence of cDCs in donor liver allografts	Acute liver allograft rejection	Yokota et al. (90)
**pDCs**			
Mouse	IL-27 production and STAT3-dependent IL-27-induced PD-L1 expression	Promote Tregs; adoptive transfer suppresses DTH responses	Matta et al. (36)
Mouse	Express high levels of DAP12/TREM2 and high PD-L1:CD86 ratios	Potently suppress allogeneic T cell proliferation; pDC-depleted donor livers rejected acutely and Treg and exhausted CD8 T cells in grafts reduced; Treg in LNs reduced	Nakano et al. (37)

Ag, antigen; ATP, adenosine triphosphate; DAP12, DNAX-activating protein of 12 kDa; DTH, delayed- type hypersensitivity; IRAK-M, interleukin-1 receptor-associated kinase M; LPS, lipopolysaccharide; LN, lymph node; MHC, major histocompatibility complex; PD-L1, programed death ligand-1; PGE2, prostaglandin E2; STAT3, signaling transducer of activated T cells; Th, T helper; TLR, Toll-like receptor; TREM2, triggering receptor of myeloid cells 2.

In the liver, the coinhibitory molecule PD-L1 is expressed constitutively by DCs, Kupffer cells and SECs (91, 92). PD-L1 also can be up-regulated on both NPCs and hepatocytes following inflammatory stimulation (55, 93–95). It has been reported that transplantation of mouse liver allografts from PD-L1 KO donors, or blocking of PD-1/PD-L1 interactions using anti-PD-L1 monoclonal antibody, results in acute liver allograft rejection. This is associated with increased graft CD8^+^ T cell infiltration and FasL perforin, granzyme B, iNOS and OPN mRNA expression in the recipients (96).

Depletion of donor interstitial DCs before mouse liver transplantation using CD11c-diptheria toxin receptor (DTR) donor mice in which DCs are depleted by DT administration, prevents induction of spontaneous allograft tolerance (90). Moreover, donor-derived cDCs can be generated *ex vivo* from progenitors present in normal mouse liver. They can also be generated from lymphoid tissue of untreated recipients of liver but not heart allografts from the same donor strain that are rejected acutely (97). In addition, when adoptively transferred to prospective pancreatic islet allograft recipients, donor liver-derived cDCs prolong graft survival (38). Collectively, these and other observations have implicated donor-derived liver cDCs in the promotion of liver transplant tolerance (70).

Absence of the transmembrane adaptor protein DAP12 (that is constitutively expressed on liver DCs at higher levels that on secondary lymphoid tissue DCs) (35) in mouse liver allografts results in higher pro-inflammatory cytokine (IL-6, IL-12p40, IFNγ, and TNFα) gene expression within the graft, enhanced IFNγ production by graft-infiltrating CD8^+^ T cells and systemic levels of IFNγ, but reduced incidences of CD4^+^Foxp3^+^ cells, associated with acute graft rejection (98).

Non-lymphoid tissue pDCs, such as those that reside in the airways, gut and liver, play a significant role in regulating mucosal immunity and are critical for the development of tolerance to inhaled or ingested/dietary Ags (99). The liver is a site of oral Ag presentation and compared to secondary lymphoid tissue, is comparatively rich in pDCs (18) that appear to rapidly induce anergy or deletion of Ag-specific T cells (37, 79). We have reported (37) that hepatic pDCs of donor origin, that express high levels of DAP12, triggering receptor of myeloid cells 2 (TREM2) and high ratios of T cell coinhibitory PD-L1:costimulatory CD86 compared with secondary lymphoid tissue pDCs, play a key role in attenuating graft-infiltrating T effector cell responses, enhancing Foxp3^+^ Tregs, and promoting spontaneous acceptance of mouse liver allografts.

Recently, we have also examined the role of graft-infiltrating DCs in regulation of mouse spontaneous liver transplant tolerance. The phenomenon of plasma membrane fragment transfer or “cross-dressing” between leukocytes was reported in 1999 (100). It has been postulated that molecules acquired by acceptor APCs during this process influence subsequent T cell responses. Several recent publications (101–103) have drawn attention to an important role of cross-dressed DCs (CD-DCs) in rejection of experimental heart, kidney and skin transplants. However, our recent novel findings (104) suggest that graft-infiltrating host cDCs that acquire donor MHC Ag shortly after liver transplantation *via* cross-dressing, regulate anti-donor T cell responses and promote allograft tolerance.

### Therapeutic Application of DCregs in Clinical Liver Transplantation

**Figure 1:**
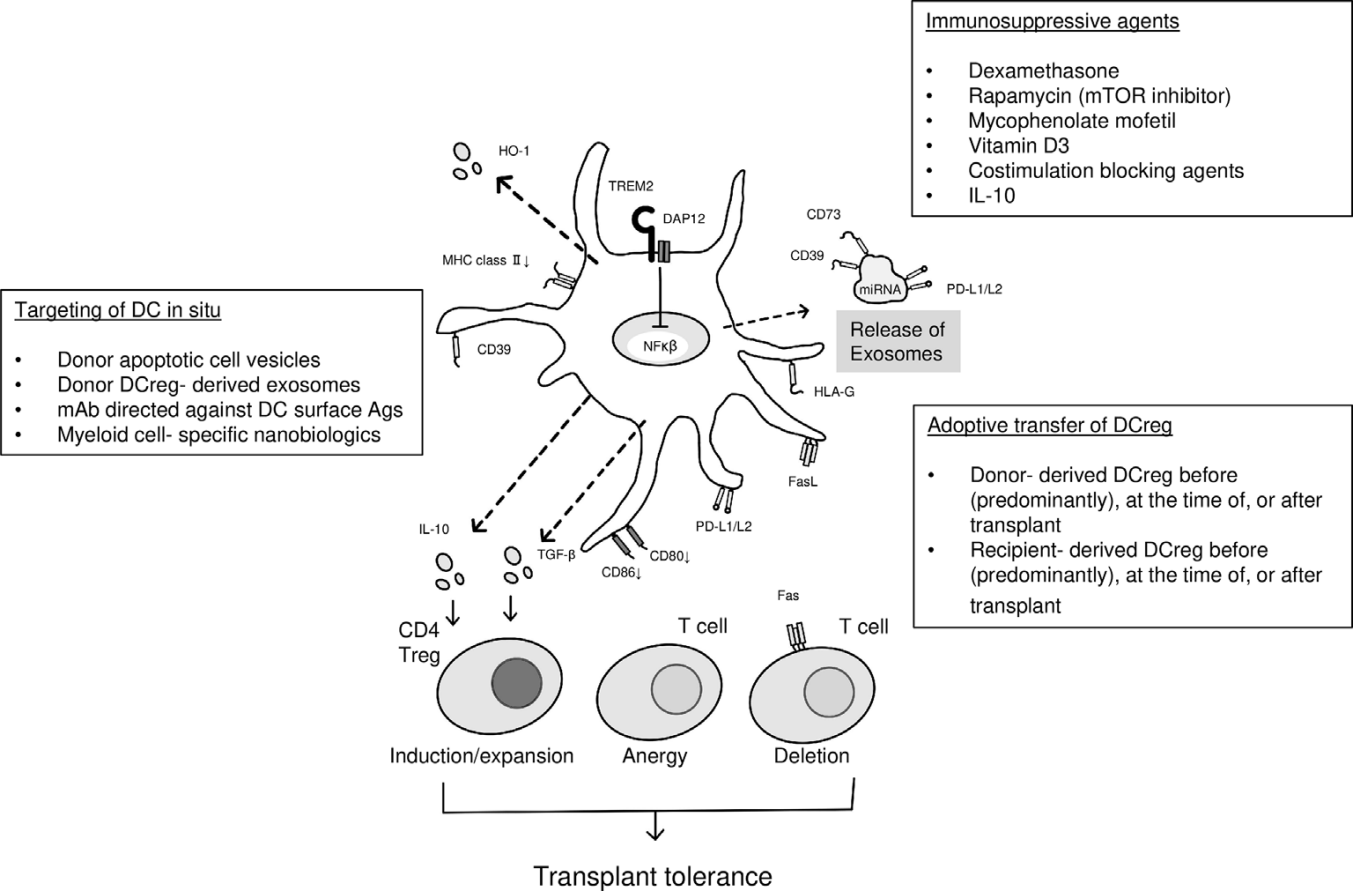
DCreg and promotion of their function. Center, DCreg showing cell membrane-expressed and secreted/molecules and released small extracellular vesicles (exosomes) that can regulate T cell responses and immune reactivity; left panel, approaches to targeting of DCreg *in situ*; upper right panel, use of immunosuppressive agents that promote DC tolerogenicity; lower right panel, adoptive transfer of DCreg in transplant recipients. DAP12, DNAX activating protein of 12 kDa; HO-1, hemoxygenase-1; PD-L1/2, programed death ligand1/2; miRNA, microRNA; TGFB, transforming growth factor beta; TREM2; triggering receptor expressed on myeloid cells 2.

Properties of DCregs and approaches to promoting their tolerogenic functions in transplantation are depicted in **Figure 1**. Following the initial observation that infusion of liver-derived cDCs, one week before transplant, could promote subsequent donor-strain allograft survival in mice (38), many rodent studies have confirmed the ability of donor-derived DCs (cDCs or pDCs) with immunoregulatory properties to enhance organ allograft survival and donor-specific tolerance (105–108). In addition, the safety and efficacy of donor-derived mDCs in prolonging MHC mis-matched renal allograft survival has been demonstrated in a clinically-relevant nonhuman primate model using a minimal IS drug regimen (109). These promising findings have provided a rationale and justification for an early phase (phase 1/2; open label, non-controlled, non-randomized) clinical trial of donor-derived DCregs in an IS drug withdrawal study in adult living donor liver transplant (LDLT) patients at the University of Pittsburgh (110).

This first-in-human study commenced in late 2017 (NCT 03164265),- donor-derived DCregs generated from circulating blood monocytes have been infused into 15 prospective liver transplant patients, once only, one week before transplantation, together with a half dose of mycophenolate mofetil (MMF) to minimize any low potential risk of host sensitization. The DCregs that are infused exhibit a tolerogenic gene transcriptional profile, high cell surface PD-L1 to CD86 ratios, secrete high levels of IL-10 but little of no IL-12 in response to TLR4- or and CD40 ligation, and only weakly stimulate proliferation of prospective graft recipient T cells (111). The dose of DCregs infused (2.5-10 x 10^6^/kg body weight) is based on the dose range that proved safe and effective in the preceding NHP studies. Patients receive conventional, post-transplant IS with steroid, MMF and tacrolimus. A protocol biopsy is performed at 1 year and, if permissive, careful weaning of tacrolimus is undertaken. Target cell numbers have been achieved for each of the prospective liver recipients and no adverse events associated with DCreg infusion have been observed. In a second clinical IS drug withdrawal study, also being performed in LDLT patients at the University of Pittsburgh (NCT04208919), a single donor-derived DCreg infusion is being administered to stable graft recipients enrolled 1-3 years post-transplant following biopsy confirmation of the absence of rejection. In addition to determining the safety of the infused DCreg product, an important objective of these studies is to determine preliminary efficacy of DCreg infusion in achieving complete IS drug withdrawal. Currently, drug withdrawal can only be achieved in 10-15% of adult liver allograft recipients in the first 2 years post-transplant (112).

### Mechanistic Studies

The initial trial of donor-derived DCregs in LDLT is being accompanied by mechanistic studies aimed at understanding the *in vivo* fate of the donor-derived DCregs and the influence of their infusion on host anti-donor immune reactivity. Following DCreg infusion one week before transplant, intact donor DCregs can be detected in host peripheral blood shortly after completion of the infusion by discriminatory MHC class I staining and flow cytometric analysis. By 3 days post infusion, no intact donor DCregs can be detected. However, in several HLA-A2 negative graft recipients given HLA-A2 positive donor cells, transiently elevated levels of both donor HLA and immunoregulatory PD-L1, CD39 and CD73 could be detected in circulating small extracellular vesicles (sEVs) (111). At the same time, flow and advanced image stream analysis revealed “cross-dressing” of host DCs in the peripheral blood and in host lymph nodes obtained at the time of surgery, before graft implantation. PD-L1 co-localization with donor HLA was observed at significantly higher levels than with recipient HLA (111). These findings resemble our observations (113) of graft-infiltrating host DCs cross-dressed with donor MHC class I Ag and co-expressing high levels of PD-L1 in mouse liver allograft recipients that accept liver allografts without IS therapy. These cross-dressed recipient DCs marked inhibited anti-donor T cell proliferation *ex vivo*. Our observations in patients also resemble the identification of circulating host APCs cross-dressed with donor MHC Ag in human liver allograft recipients early and transiently after transplantation (114). In our studies, between the time of donor DCreg infusion and liver transplantation, memory CD8^+^ T cells expressing high levels of the transcription factors T-bet and Eomesodermin (T-bet^hi^Eomes^hi^) decreased, whereas regulatory (CD25^hi^CD127^-^Foxp3^+^):T-bet^hi^Eomes^hi^ CD8^+^ T cell ratios increased. Although the number of observations is small, this increase appeared to be associated with the incidence of cross-dressed DCs observed in the circulation. Thus, it appears that donor-derived DCreg infusion in prospective liver transplant recipients may induce systemic changes in host APCs and T cells that may be conducive to modulated anti-donor immune T cells responses at the time of transplantation. We postulate that the composition (quality) of sEVs rather than the density (quantity) of peptide MHC-expressing sEVs on cross-dressed DC may play an important role in the induction of peripheral tolerance.

## Conclusions

Liver interstitial DCs appear to play important roles in the regulation of hepatic IRI and other inflammatory responses within the liver environment. Donor-derived DCs and more recently, graft-infiltrating host DCs that have acquired intact donor MHC Ag *via* cross-dressing, have been implicated in the promotion of spontaneous liver transplant tolerance in the mouse. Demonstrations that adoptive transfer of donor-derived DCregs can prolong organ transplant survival and tolerance in preclinical models has led to clinical testing of DCregs for promotion of transplant tolerance in human liver transplantation. These studies are accompanied by mechanistic investigations designed to enhance insight into the influence of these cells on host anti-donor immune reactivity.

## Author Contributions

RN: drafting of the manuscript. LT, DG, CM, and DM: critical review of the manuscript. AT: concept, design and writing of the manuscript. All authors contributed to the article and approved the submitted version.

## Funding

The authors’ work is supported by National Institutes of Health (NIH) grants R01 AI118777, U01 AI136779 and U19 AI131453 and by the Immune Transplant and Therapy Center of the University of Pittsburgh Medical Center. LT is supported by NIH institutional research training grant T32 AI074490 and the Physician-Scientist Institutional Award from the Burroughs Wellcome Fund held by the University of Pittsburgh.

## Acknowledgments

We thank Dr Masahiko Kubo for help in generation of **Figure 1**.

## Conflict of Interest

The authors declare that the research was conducted in the absence of any commercial or financial relationships that could be construed as a potential conflict of interest.
